# Docking studies on novel analogues of 8 methoxy fluoroquinolones against GyrA mutants of *Mycobacterium tuberculosis*

**DOI:** 10.1186/1472-6807-11-47

**Published:** 2011-12-12

**Authors:** RS Anand, Sulochana Somasundaram, Mukesh Doble, CN Paramasivan

**Affiliations:** 1 Centre for Biotechnology, Anna University, Chennai 600 025, India; 2 Department of Biotechnology, Sri Venkateswara College of Engineering, Sriperumbudur 602 105, India; 3 Department of Biotechnology, Indian Institute of Technology - Madras, Chennai 600 036, India; 4 Foundation for Innovative New Diagnostics, 16, Avenue de Budé 1202 Geneva, Switzerland

**Keywords:** Docking, *Mycobacterium tuberculosis*, GyrA, Fluoroquinolones, gatifloxacin, moxifloxacin

## Abstract

**Background:**

Fluoroquinolone resistance is a serious threat in the battle against the treatment of multi drug resistant tuberculosis (MDR-TB) and extensively drug resistant tuberculosis (XDR-TB). Fluoroquinolone resistant isolates from India had shown to have evolved several mutants in the quinolone resistance determining region (QRDR) of DNA gyrase A subunit (GyrA), the target of fluoroquinolone. In view of high prevalence of mutations in the 'hot spot' region, a study on combinatorial drug design was carried out to identify better analogues for the treatment of MDR-TB. The *gyrA *subunit 'hot spot' region of codons 90, 94 and 95 were modeled into their corresponding protein folds and used as receptors for the docking studies. Further, invitro tests were carried using the parent compounds, namely gatifloxacin and moxifloxacin and correlated with the obtained docking scores.

**Results:**

Molecular docking and *in vitro *studies correlated well in demonstrating the enhanced activity of moxifloxacin, when compared to gatifloxacin, on ofloxacin sensitive and resistant strains comprising of clinical isolates of MDR-TB. The evolved lead structures targeting against mutant QRDR receptors were guanosine and cholesteryl esters of gatifloxacin and moxifloxacin. They showed consistently high binding affinity values of -10.3 and -10.1 kcal/mol respectively with the target receptors. Of these, the guanosine ester showed highest binding affinity score and its log P value lied within the Lipinski's range indicating that it could have better absorptivity when it is orally administered thereby having an enhanced activity against MTB.

**Conclusions:**

The docking results showed that the addition of the cholesteryl and guanosine esters to the 'DNA gyrase binding' region of gatifloxacin and moxifloxacin enhanced the binding affinity of these parent molecules with the mutant DNA gyrase receptors. Viewing the positive correlation for the docking and in vitro results with the parent compounds, these lead structures could be further evaluated for their *in vitro *and *in vivo *activity against MDR-TB.

## Background

The resurgence of multi-drug resistant tuberculosis (MDR-TB) [1] and HIV associated intractable mycobacterial infection are of serious global concern [2]. To contain this situation, new anti-tuberculosis drugs and reduced regimen treatments are of immediate requirement. Development of novel antituberculosis compounds to combat MDR-TB is urgently needed. Unfortunately, except for rifabutin and rifapentine there are no new drugs available during the 40 years after the release of rifampicin. The discovery of new drugs involves several constraints that discourage many companies from investing in novel anti-TB drugs. The research is expensive, slow and difficult, and it requires specialized facilities for handling *Mycobacterium tuberculosis *(MTB). Due to this situation, it is a matter of urgency to develop other new anti-tuberculous drugs, especially with the aid of bioinformatics-based drug design, in order to tackle intractable TB [3]. The 8-methoxy quinolones are highly efficacious in showing potent anti-TB therapeutic activities in humans and animals, particularly when added to multidrug regimens, [4,5] suggesting their usefulness as first-line drugs for TB. They can also be used for the treatment of proven MDR-TB, for the empirical treatment of TB in settings of high rates of MDR-TB and for patients with severe adverse reactions to ordinary first-line drugs [6]. These fluoroquinolones bind to DNA gyrase thereby stabilizing the covalent intermediate of DNA super coiling [7]. Currently they are used excessively in the treatment against MDR-TB and XDR-TB [8]. Frequent usage of these fluoroquinolones lead to resistance towards these drugs. Missense mutations in the putative fluoroquinolone binding region of the A subunit has been found to confer high level resistance and is referred to as QRDR. The mutations in codons, 90 (Arg to val), 94 (Asp to Asn, Gly) and 95 (Ser to Thr, Ala and Val), of *gyrA *gene are found in the drug resistant strains of MTB [9]. But the new derivatives, moxifloxacin (MFX) and gatifloxacin (GFX) are found to have more efficient activity towards fluoroquinolone resistant MTB with a low mean MIC. Hence, modern drug designing techniques including quantitative structure activity relationship (QSAR) [10], Molecular Docking etc. are widely used to understand the structural features of compounds responsible for pharmacological activities

**Figure 1:**
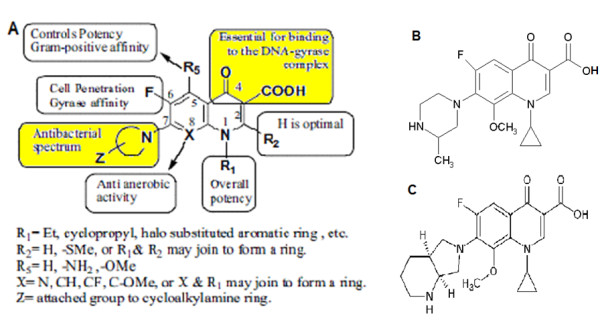
**Common structural features of quinolones**. (A) The modifications were made at positions 3 and 7, essential for binding to DNA-gyrase complex and antibacterial spectrum respectively [20]. (B) and (C) are the chemical structures of gatifloxacin and moxifloxacin respectively.

In our study, the mutant GyrA molecules were modeled as their crystal structures are not yet available and a suitable antagonist design was carried out using molecular docking. The antagonist design was carried out with the modifications involving hydrophilic and lipophilic moieties. Hydrophilic moieties include Amino (NH_2_), Hydroxyl (OH), Fluorine (F), phosphate, lactone, glucose and esters of Adenine, Thymidine, Guanine and Cytidine. While lipophilic moieties include benzene, napthalene, phenanthacene, saturated lipid tails and cholestryl esters. All these modifications were made at positions 3 and 7 in the fluoroquinolone structure (Figure 1A) to identify analogues which may exhibit better therapeutic efficacy against MDR-TB [11]. These positions contribute to the DNA gyrase binding and broad spectrum antibacterial activity properties respectively. In addition, the theoretically calculated binding affinities of gatifloxacin and moxifloxacin were compared with their MICs to have a practical correlation for the docking studies.

## Methods

### Strain selection

The resistance pattern of 39 isolates of *M. tuberculosis *isolated from chronically ill, sputum smear positive patients with a history of previous anti tuberculosis treatment received from various parts of India formed the basis of this study. Of these 39 strains, 17 were susceptible to ofloxacin, which comprised of 11 MDR strains (resistant at least to INH-isoniazid and RMP- rifampicin), one strain resistant to INH alone, while the remaining 5 susceptible to all drugs. Of the 22 ofloxacin (OFX)-resistant strains, 21 were MDR strains and 1 was resistant to INH alone. The drug susceptibility testing for GFX and MFX was done by Absolute concentration method on Lowenstein Jensen medium for various concentrations as described Sulochana *et al.*, [12] and the results were used in this study.

### Mutation identification of *gyrA*

**Figure 2:**
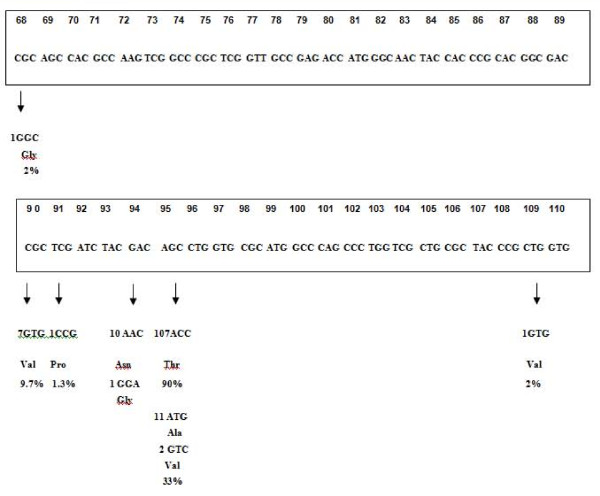
**Mutations in the QRDR of gyrA gene of* M. tuberculosis* clinical isolates**. The mutated codons and the corresponding amino acids changes are shown at the bottom. The original sequence is shown in the box. Numbers indicate the number of isolates showing the mutations, while the percentage denote the frequencies of occurrence of mutations at the particular codon [11].

Mutations present in the above strains (Figure 2) were identified by sequencing PCR amplified product of *gyrA *using automated sequencer ABI Prism model 377 version 10.0 with Big dye terminator. The data obtained were compared with the sequence available at the Sanger Center and NCBI using BLAST program [13] and the mutational pattern was compared with the phenotypic susceptibility pattern [14].

### Docking tools

The binding affinity of the analogues were obtained using AUTODOCK Vina tool with AMBER force field and Monte Carlo simulated annealing [15]. The dockings were performed in a 64 bit PC. The receptor design was made by using SWISS-MODEL, a fully automated protein structure homology-modeling server. In this tool, energy minimization and simulated annealing are done with the GROMOS96 forcefield [16]. The 2 D structures of the ligands were drawn, optimized with full hydrogen bonds and saved as. sk2 format using ChemSketch tool from Advanced Chemistry Development, Inc. [17] and the 3 D structures were obtained using PRODRG server [18].

### Receptors

Four "Hot spot" mutations (90 GTG, 94 AAC, 95 ATG and 95 ACC) in the QRDR of GyrA subunit (Figure 2) expressed in 7, 10, 11 and 107 in the clinical MTB isolates respectively as described by Sulochana *et al *[14] were used for modeling the protein receptor. The GyrA crystal structure (PDB: 3IFZ) elucidated by Piton et al., [19] was used for designing the mutant receptors. The SWISS-MODEL receptor design was built from the 2.00 Å crystal structure coordinates of 25-kDa periplasmic His/Glu/Gln/Arg/opine family-binding protein from *Silicibacter pomeroyi *(PDB: 3L6V) since the available wild type GyrA crystal structure (PDB: 3IFZ) based model building was not successful for the mutated sequences due to poor sequence alignment.

### Ligands

The developed ligands using the ChemSketch tool were based on the structures of GFX (Figure 1B) and MFX (Figure 1C) since these drugs have better activity, both *in vitro *[20,21] and *in vivo *against MDR-TB [22]. Various modifications in the 'Antibacterial spectrum' determining region and 'DNA gyrase binding' region of the fluoroquinolone as mentioned by Emami *et al.*, [23]. The ligands were designed using the structures of GFX and MFX by using lipophilic and hydrophilic moieties at positions 3 and 7, essential for binding to DNA-gyrase complex and antibacterial spectrum respectively (Additional file 1, Figure S1).

### Analysis of Binding

**Figure 3:**
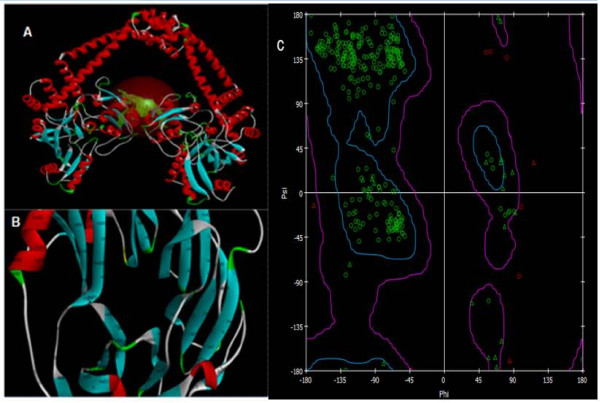
**Structural view of receptors and their optimization**. (A) PDB image of *Mycobacterium tuberculosis* DNA gyrase A (PDB: 3IFZ). (B) Modeled third mutant receptor corresponding to the QRDR of GyrA. (C) Ramachandran Plot for the third mutant receptor after energy optimization.

The binding sites for the docking were designed such that the entire receptor molecule was included within the selection grid. The highest binding energy values corresponding to the RMSD value of zero were considered as the binding affinity value of the ligands for each docking. The Hydrogen bond interactions were obtained using Molegro molecular viewer [24] and Ramachandran plot (Figure 3) obtained from Discovery studio 3.1 Visualizer [25] was used to confirm the integrity of the designed protein fold.

## Results

MIC values for MFX, GFX and OFX on various strains of *Mycobacterium tuberculosis *comprising of four mutant codons in QRDR region of GyrA were given (Table 1). The binding scores were calculated by using AUTODOCK Vina tool and the binding affinity was compared with their binding scores.

**Table 1:** MIC values of Moxifloxacin, gatifloxacin and ofloxacin on various strains of *Mycobacterium tuberculosis *comprising the four mutant codons in QRDR region of *gyrA*.

Strain data	**S. No**.	**Strain No**.	MDR status	Resistance to Ofloxacin	Area	MFX (μg/ml)	GFX (μg/ml)	OFX (μg/ml)
First mutant - Codon 90 Ala-Val	1	944284	MDR	R(M)	Goa	5	5	8

	2	940228	MDR	R(L)	Chennai	NA	NA	32

**Binding affinity (kcal/mol)**						**-7.9**	**-7.3**	**-7.4**

Second mutant - Codon 94 Asp-Asn	3	936837	MDR	R(H)	Sikkim	NA	NA	64

**Binding affinity (kcal/mol)**						**-7.9**	**-7.3**	**-7.4**

Third mutant - Codon 94 Asp-Ala	4	912607	MDR	R(H)	Chennai	NA	NA	64

	5	938424	MDR	R(H)	Karnataka	5	5	16

	6	937532	MDR	R(M)	Chennai	2	5	8

	7	939784	MDR	R(M)	Chennai	> 5	2	32

	8	940183	MDR	R(L)	Sikkim	NA	NA	16

	9	RF0048	MDR	R(H)	NA	5	5	16

**Binding affinity (kcal/mol)**						**-7.9**	**-7.4**	**-7.1**

Fourth mutant - Codon 95 Ser-Thr (Natural polymorphism)	10	910659	MDR	S	Chennai	0.25	1	2

	11	913168	MDR	R(L)	Chennai	NA	8	16

	12	916590	MDR	R(M)	Tamil Nadu	NA	8	32

	13	929925	MDR	R(L)	Chennai	1	1	8

	14	932503	MDR	S	Chennai	0.5	0.5	2

	15	933750	MDR	R(H)	NA	5	5	8

	16	932504	MDR	R(L)	Chennai	2	2	8

	17	937583	MDR	R(L)	Chennai	1	2	16

	18	937911	MDR	R(H)	UP	> 5	> 5	32

	19	938066	Non MDR	S	Chennai	NA	NA	4

	20	939184	MDR	R(L)	Chennai	NA	NA	8

	21	939271	MDR	S	Chennai	NA	NA	4

	22	940586	MDR	R(H)	AP	5	> 5	64

	23	940742	Non MDR	S	Chennai	1	0.5	4

	24	941935	INH resistant RMP sensitive	R(L)	Chennai	NA	NA	8

	25	942100	MDR	R(L)	Chennai	5	5	16

	26	947356	MDR	R(M)	Chennai	5	2	32

	27	RF0071	MDR	R(L)	NA	NA	NA	8

	28	939825	MDR	S	Chennai	1	1	4

	29	939850	Non MDR	S	Chennai	0.25	0.5	2

	30	940031	MDR	S	Chennai	0.25	0.5	4

	31	940033	INH resistant RMP Sensitive	S	Chennai	0.5	0.5	2

	32	RF0057	Non MDR	S	NA	NA	NA	2

	33	RF0095	MDR	S	NA	0.5	0.2	2

	34	RF0096	MDR	S	NA	0.125	0.2	2

	35	RF0119	MDR	S	NA	NA	NA	2

	36	RF0130	MDR	S	NA	0.25	0.2	2

	37	RF0131	MDR	S	NA	0.25	0.5	2

	38	949998	Non MDR	S	Chennai	1	0.5	4

	39	950629	MDR	S	Chennai	1	0.5	2

**Binding affinity (kcal/mol)**						**-7.9**	**-7.3**	**-7.6**

Wild type (H37 Rv)						0.25	0.25	< 2

**Binding affinity (kcal/mol)**						**-7.9**	**-7.4**	**-7.6**

### MIC of MFX vs Binding affinity scores

The MIC value of moxifloxacin to the first mutant receptor (codon 90 GTG) containing strain (Lab No.944284) was 5 μg/ml and the binding affinity was -7.9 kcal/mol. The same binding affinity value was also observed for the second mutant receptor (codon 94 AAC) of OFX resistant strain. All the four ofloxacin resistant strains with third mutant receptor (codon 94 ATG), showed MIC values between 2 to > 5 μg/ml for MFX and a same binding affinity score of -7.9 kcal/mol. The fourth mutant receptor (codon 95 ACC), considered as a natural polymorphism was found in 13 resistant and 17 sensitive strains of ofloxacin. These 13 ofloxacin resistant strains which comprised of 12 MDR and one INH mono resistant strain had the MIC for moxifloxacin ranging from 1 to > 5 μg/ml with the same binding affinity score of -7.9 kcal/mol. Of the 17 ofloxacin sensitive strains which comprised of 11 MDR, 5 Non MDR and 1 INH monoresistant strains, the MIC for moxifloxacin was between 0.125 to 1 μg/ml with the binding affinity score of -7.9 kcal/mol. Irrespective of the sensitivity pattern for ofloxacin and MDR status, the binding affinity values for moxifloxacin, remained the same in all cases (Table 1).

### MIC of GFX vs Binding affinity scores

Considering the first mutant receptor (codon 90 GTG) containing strains, the MIC value of moxifloxacin to the strain (Lab No.944284) was 5 μg/ml and the binding affinity was -7.3 kcal/mol. The same binding affinity value was also observed for the second mutant receptor (codon 94 AAC) of ofloxacin resistant strain. With respect to the third mutant receptor (codon 94 ATG), all the four ofloxacin resistant strains showed the MIC values between 2 to > 5 μg/ml for gatifloxacin with an increased binding affinity score of -7.4 kcal/mol. The fourth mutant receptor (codon 95 ACC), considered as a natural polymorphism was found both in 17 sensitive and 13 resistant strains of ofloxacin. Of 13 ofloxacin resistant strains which comprised of 12 MDR and one INH mono resistant strain had the MIC for gatifloxacin ranging from 2 to > 5 μg/ml. Of the 17 ofloxacin sensitive strains which comprised of 11 MDR, 5 Non MDR and 1 INH monoresistant strains, the MIC of gatifloxacin ranging from 0.2 to 1 μg/ml. This mutant receptor showed a binding affinity value of -7.3 kcal/mol (Table 1).

### Interaction of MFX and GFX with the binding site

**Figure 4:**
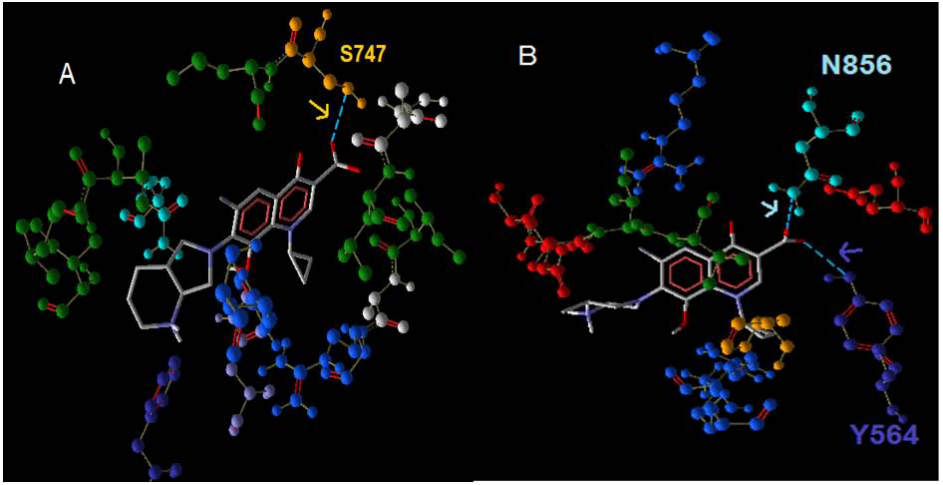
**Interactions of Moxifloxacin and Gatifloxacin with the third mutant receptor**. The dotted light blue line shows the H-bond interactions of Moxifloxacin (A) and Gatifloxacin (B). The H- bond interactions are indicated by arrow marking in the colour of the corresponding amino acid.

**Figure 5:**
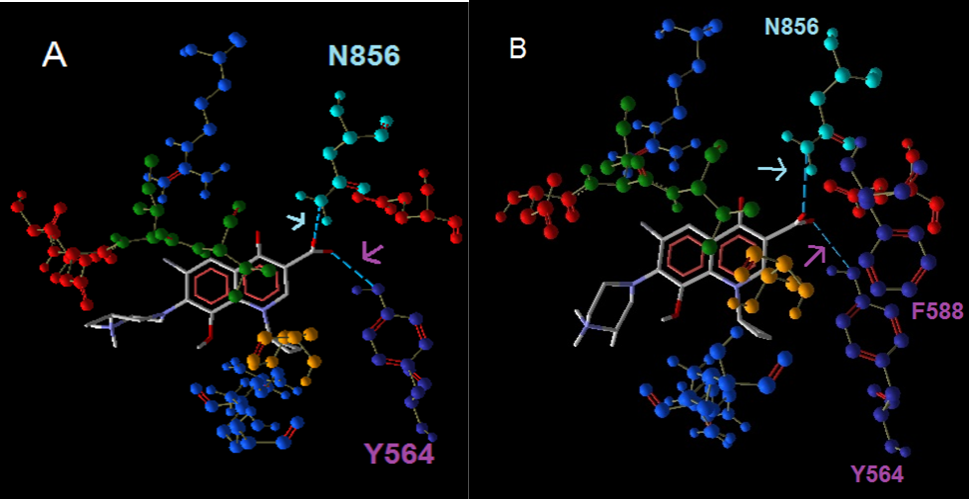
**Interactions of Gatifloxacin with the second and third mutant receptor**. The dotted light blue line shows the H-bond interactions of gatifloxacin with third (A) and second mutant receptors (B). The H- bond interactions are indicated by arrow marking in the colour of the corresponding amino acid.

The interaction of MFX and GFX with the GyrA binding site showed that they interacted with different amino acid residues at the active site (Figure 4). Only a single H-bonding between the carboxyl oxygen and the hydroxyl group of serine747 existed for moxifloxacin whereas gatifoxacin showed two potential H-bond interactions with asparagine 856 and tyrosine 564. But considering the high binding affinity value of moxifloxacin (-7.9 kcal/mol) when compared to that of gatifloxacin (-7.4 kcal/mol), as observed by Kitchen *et al.*, [26] it could be attributed to other bonding forces. The interaction of gatifloxacin with the second and third mutant GyrA binding site showed that with both the receptors, the H-bond interactions occurs with the Tyrosine 564 and Asparagine 856. The H-bond energy between the carboxyl group of gatifloxacin and the oxygen atom of tyrosine yielded the -2.5 kcal/mol for the third mutant receptor whereas it showed lower value of -1.411 kcal/mol for the second mutant receptor in the presence of non interacting phenylalanine 588. This may explain the net lower affinity value of the second mutant receptor (-7.3 kcal/mol) with respect to the third mutant receptor (-7.4 kcal/mol) (Figure 5).

### MIC vs Binding affinity correlation

**Figure 6:**
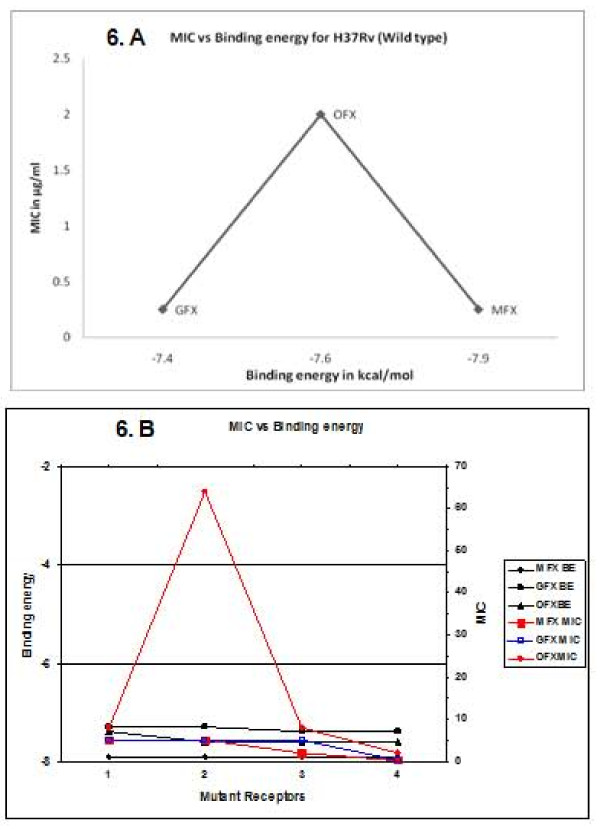
**Comparison between binding energy and MIC**. (A) Comparison of the docking scores with MIC of GFX, OFX and MFX for the wild type. (B) Comparison of the docking scores with MIC of MFX, GFX and OFX for the mutants.

The graphical representation of the binding affinity of gatifloxacin and moxifloxacin with all the four different mutants and wild type receptors showed relatively greater binding affinity for moxifloxacin than gatifloxacin (Figures 6A and 6B). When comparing to the MICs, GFX and MFX showed better values for 7 and 8 out of 26 strains (for which the MIC values of both MFX and GFX were determined) respectively (Table 1). Their values remained equal for the remaining strains. Considering the graphical representation of MIC for MFX, it can be seen that MFX is having a comparable bactericidal activity to GFX with first, second and fourth mutant receptors whereas it had enhanced activity with the third mutant receptor. The binding energy shows a uniform improved activity for MFX when compared to GFX. The large peak in the Figure 6B is because of the poor activity of OFX (64 μg/ml) for the second mutant when compared to GFX and MFX.

### Docking analogues

In order to improve the binding affinity, two different modifications on the 'Antibacterial spectrum' determining region and 'DNA gyrase binding' region, as described by Emami *et al.*, [20] were attempted in the structures of GFX and MFX. The analogues are listed in Additional file 1, Figure S1.

### Modifications to 'Antibacterial spectrum' determining region modifications

**Figure 7:**
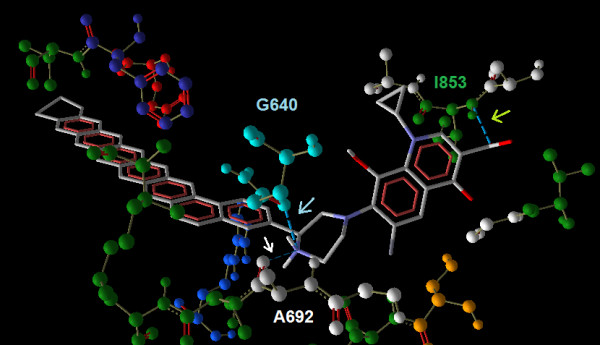
**Binding of Ligand 19 with the binding site of third mutant receptor**. The H- bond interactions are indicated by arrow marking in the colour of the corresponding aminoacid.

Increasing the aromaticity in this position showed a marked increase in the binding affinity values ranging from -7.9 (for Ligand 1) to -12.3 kcal/mol (for Ligand 19) with all the four mutants and wild type receptors. Ligand 19 with seven aromatic and one cyclo hexane moieties attached in this position showed the highest binding affinity, a value of -12.3 kcal/mol for all the four mutant receptors (Table 2 and Figure 7). This indicated that the mutations in the binding site did not affect the binding affinity of this ligand. Binding site of the third mutant receptor, with the ligand 19, showed three hydrogen bond interactions with Glycine 640, Alanine and Isoleucine 853 (Figure 7). The strongest interaction was found between the Nitrogen atom at the 7^th ^position of the piperazine ring and glycine, with a H-bond energy of -2.5 kcal/mol and a bond length of 3.041 nm. From the interactions it can be seen that the rigid aromatic side chain form a stacking effect in presenting the interacting domain to the binding site. The net interactions yielded a highest binding affinity (of -12.3 kcal/mol) for this compound with this receptor (Table 2). On the other hand, when this structure was modified so as to replace the aromaticity with a saturated lipid tail (Ligand 20), interestingly it showed a drastic decrease in the binding affinity with all the four mutant receptors namely -7.2, -6.1, -6.7 and -6.6 kcal/mol respectively, which were the lowest binding affinity values when compared to all other ligands (Table 2).

**Table 2:** Docking scores of gatifloxacin, moxifloxacin and their new derivatives with respect the four mutant and the wild type receptors

**Ligand No**.	First mutant Codon 90 Ala -Val	Second mutant Codon 94 Asp-Asn	Third mutant Codon 94 Asp-Ala	Fourth mutant Codon 95 Ser-Thr	Wild type
Ciprofloxacin	-7.5	-7.5	-7.4	-7.5	-7.5

Gatifloxacin	-7.3	-7.3	-7.4	-7.3	-7.4

Lomefloxaciin	-7.6	-7.6	-7.6	-7.6	-7.5

Moxifloxacin	-7.9	-7.9	-7.9	-7.9	-7.9

Ofloxacin	-7.6	-7.6	-7.6	-7.6	-7.6

Sparfloxacin	-8.0	-8.0	-8.1	-8.1	-8.1

1	-8.0	-8.2	-8.2	-7.9	-8.2

2	-8.4	-8.5	-8.4	-8.4	-8.5

3	-8.1	-8.1	-8.5	-8.1	-8.1

4	-8.3	-8.1	-8.3	-8.4	-8.1

5	-8.2	-8.4	-8.4	-8.2	-8.5

6	-9.0	-9.0	-9.0	-9.0	-9.0

7	-9.2	-9.1	-9.2	-9.2	-9.1

8	-10.1	-10.0	-10.1	-10.1	-10.1

9	-9.5	-9.5	-9.5	-9.5	-9.5

10	-9.6	-9.7	-9.7	-9.7	-9.7

11	-9.5	-9.5	-10.0	-9.5	-9.5

12	-10.8	-10.8	-10.8	-10.7	-10.7

13	-10.2	-10.2	-10.2	-9.8	-10.2

14	-7.6	-7.5	-7.5	-7.5	-7.6

15	-7.9	-7.9	-7.9	-7.9	-7.9

16	-11.7	-11.7	-11.7	-11.7	-11.7

17	-11.4	-11.5	-11.5	-11.5	-11.5

18	-12.1	-12.1	-12.1	-12.1	-12.1

19	-12.3	-12.3	-12.3	-12.3	-12.0

20	-7.2	-6.1	-6.7	-6.6	-6.6

21	-8.4	-8.4	-8.4	-8.4	-8.4

22	-8.7	-8.7	-8.7	-8.7	-8.7

23	-8.9	-8.9	-8.9	-8.9	-8.9

24	-7.1	-7.1	-7.1	-7.1	-7.1

25	-8.6	-8.6	-8.6	-8.5	-8.6

26	-8.1	-8.7	-8.7	-8.7	-8.6

27	-9.0	-9.0	-9.2	-9.0	-9.1

28	-8.8	-9.7	-9.2	-9.7	-9.2

29	-8.8	-9.2	-9.2	-9.0	-9.0

30	-9.7	-9.0	-8.9	-9.0	-8.8

31	-8.9	-9.5	-9.3	-9.3	-9.5

32	-8.5	-8.7	-8.9	-9.0	-8.9

33	-9.4	-9.8	-9.4	-9.4	-9.4

34	-9.0	-9.0	-8.7	-9.0	-8.6

35	-10.3	-10.3	-10.3	-10.4	-10.3

36	-10.1	-10.0	-10.1	-9.8	-10.1

37	-9.0	-9.2	-9.2	-9.2	-9.2

38	-8.2	-8.0	-7.9	-7.7	-8.0

39	-8.6	-8.6	-8.6	-8.6	-8.6

### Modifications to 'DNA gyrase binding' region

**Figure 8:**
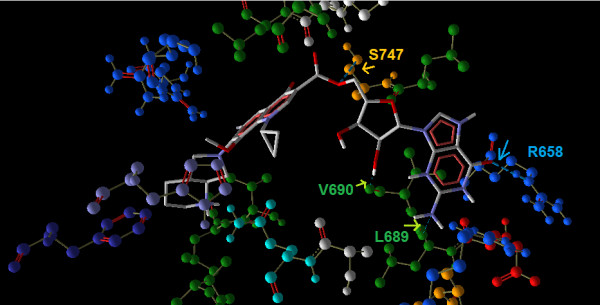
**Binding of Ligand 35 with the binding site in the third mutant receptor**. The H- bond interactions are indicated by arrow marking in the colour of the corresponding amino acid.

The modifications to the 'DNA gyrase binding' region mainly consisted of esters at the carboxyl site. The modifications at this site yielded docking scores ranging from -7.7 (Ligand 38) to 10.4 (Ligand 35). With respect to the nucleoside esters, Ligand 35, the guanosine ester of moxifloxacin, showed the highest binding score namely, -10.3 kcal/mol for first, second, third and wild type receptors. Whereas for the fourth mutant receptor it showed an enhanced score of -10.4. The increased affinity of this ligand when compared to the other nucleoside esters can be correlated to the four hydrogen bond interactions it formed with Serine 747, valine 690, Leucine 689 and arginine 658 (Figure 8). The strongest interaction was found between the ester oxygen and hydroxyl group of serine 747 with a H-bond energy of -2.5 kcal/mol and a bond length of 2.83 nm. The net interactions yielded a binding affinity of -10.3 kcal/mol for this compound with this receptor (Table 2).

**Figure 9:**
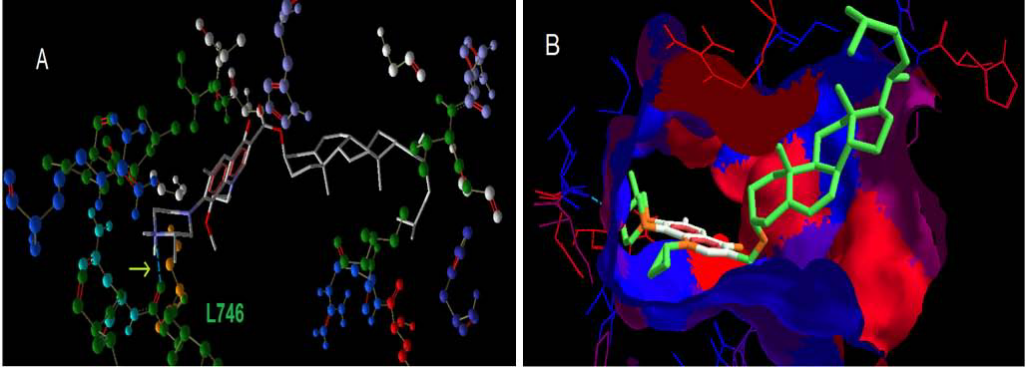
**Binding of Ligand 36 with the binding site of first mutant receptor**. (A) The H- bond interactions are indicated by arrow marking in the colour of the corresponding aminoacid. (B) Shows the orientation of ligand 36 with the hydrophobic surfaces (Red) and hydrophilic surfaces (blue) in the binding pocket of the first mutant receptor.

Endogeneous ester modification of gatifloxacin, Ligand 36, which had a cholesteryl ester at this position, yielded a reasonably good docking score (-10.1 kcal/mol) with respect to first, third and wild type receptors (Table 2). This good binding affinity arises from the H bond interaction between the nitrogen atom in the 39^th ^position in the structure and the carbonyl oxygen of Leucine 746. The energy value of this interaction was -2.5 kcal/mol and a bond length of 2.97 nm (Figure 9A). The docked image of ligand 36 in the binding pocket of GyrA showed high hydrophobic surface interactions which could also contribute to the observed high binding affinity (Figure 9B).

From the results obtained, it could be concluded that the binding affinity of moxifloxacin with that of the wild type and all the four mutant receptors were similar with a uniform affinity value (-7.9 kcal/mol). Whereas, for gatifloxacin the binding affinity was -7.3 kcal/mol with the first, second and fourth mutant receptors and a relatively higher score of -7.4 kcal/mol with the third and wild type receptors. The high binding affinity scores obtained for moxifloxacin when compared to that for GFX also correlates with their MIC values. Further, ligand 36 and 35, with cholestryl and guanosine esters respectively showed to be leading structures with good binding affinity scores.

## Discussion

### Comparison between moxifloxacin and gatifloxacin for resistant strains

Twenty nine strains of *Mycobacterium tuberculosis *showed S-T polymorphism which did not code for any resistance pattern but occurred as natural polymorphism [27] were identified. Of these, 13 ofloxacin resistant strains with no mutations showed phenotypic resistance pattern which might be due to the efflux mechanisms observed in *M. smegmatis *[28]. These resistant strains showed high sensitivity to both moxifloxacin and gatifloxacin (Figure 6). Our earlier *in vitro *findings on actively growing and persister *Mycobacterium tuberculosis *H37 Rv, showed that moxifloxacin is considered to have good sterilizing activity in addition to early bactericidal activity [20]. This could be correlated with the higher binding affinity calculated between the mutant and wild type Gyr A receptors and moxifloxacin (-7.9 kcal/mol) when compared to the maximum binding affinity output from gatifloxacin (-7.4 kcal/mol) (Table 1). This could also be extended to the observation that although gatifloxacin exhibited good bactericidal activity against multiplying organisms it had very limited sterilizing activity against persisters [20].

### Evaluation of lead structural analogues

The high docking score of cholestryl ester with all the four mutant receptors can be due to the hydrophobicity of the binding pocket of GyrA (Figure 9B). Guzman *et al.*, [29] showed that a cholesterol-rich diet accelerated the sterilization rate of sputum cultures in pulmonary tuberculosis patients, suggesting that cholesterol should be used as a complementary measure in antituberculous drug treatment. Study by Brzostek *et al.*, [30] also showed that cholesterol oxidase (ChoD), a well-known cholesterol modification enzyme, is important for the virulence for the tubercle bacillus. The theoretical purpose of having a cholesterol moiety in the modified fluoroquinolones in the form of esters is that it enables the drug to act as a prodrug such that its active portion is released in the presence of ChoD. However, the mechanism of MTB uptake by mast cells is not known. Munoz *et al.*, [31] showed that *Mycobacterium tuberculosis *(MTB) enters into the mast cells through cholesterol-enriched membrane microdomains (lipid rafts). This supports our hypothesis that cholesterol conjugated fluoroquinolones could have better penetrating efficiency towards the intracellular MTB. This is also evidenced by a study carried out by Mohammed *et al.*, [32] who showed that the cholesterol content of the liposome bilayer influenced the incorporation efficiency of ibuprofen to the maximum. Schmalfuß *et al.*, [33] showed that cholesterol increased the drug penetration in the tissues. This is further supported with the evidence showed by Simoes *et al.*, [34], wherein lipophilic ester prodrugs of Pyrazinamide (PZA) were found to be active in concentrations 10-fold lower than those needed for PZA.

In this study, the esters of nucleosides namely, adenine, thymidine, guanine and cytosine were attempted since the active site of the DNA gyrase has highly conserved complementary residues for binding with the single stranded DNA so as to prevent it from supercoiling. This binding of DNA gyrase must be predominantly involved in the interactions with the bases in the DNA strand. This might support the enhanced binding affinity of the modified drug containing nucleoside esters which pairs with complementary bases in the single stranded DNA. This nucleotide ester might also act by blocking DNA replication. This type of mechanism is similar to that approached for nucleoside analogues such as Ribavirin, an antiviral agent. Its carboxamide group can make the native nucleoside drug resemble like adenosine or guanosine, depending upon its rotation. So, when ribavirin is incorporated into RNA, as a base analog of either adenine or guanine, it pairs equally well with either uracil or cytosine, inducing mutations in RNA-dependent replication in RNA viruses [35]. Earlier studies by Huang *et al.*, showed that tyrosine 577, arginine 691 and arginine 745 are among the key DNA-binding residues in *M. tuberculosis *DNA gyrase A subunit [36]. In this study, interestingly, GFX interacted with tyrosine 564, MFX interacted with serine 747, guanosine ester of moxifloxacin (Ligand 35) formed H bond interactions with Leucine 689, valine 690 and serine 747 moreover cholestryl ester of gatifloxacin (Ligand 36) also showed H bond interaction with leucine 746, all of which are close to the key DNA-binding residues identified by Huang *et al.*

In summary, the docking results showed that the addition of the cholesteryl and guanosine esters to the 'DNA gyrase binding' region of gatifloxacin and moxifloxacin enhanced the binding affinity of the parent molecule. *M. tuberculosis *invades the macrophages via the cholesterol dependent pathway [31] and this supports the hypothesis that the lead cholestryl ester compound could have a similar activity as that of pyrazinamide at acidic pH against intracellular MTB [37]. The guanosine ester showed highest binding affinity score and its log P lies between value of -0.45 and 2.69, which is within the Lipinski's range of 1 to 5 indicating that it could have good absorptivity when it is orally administered thereby having an enhanced activity against MTB.

## Conclusion

The docking scores confirmed our earlier *in vitro *studies that moxifloxacin had a better killing activity than gatifloxacin against *Mycobacterium tuberculosis *H37 Rv [20,21]. The docking studies also yielded that increase in the lipophilicity on either of the positions of the quinolone structure considerably increased the binding affinities. But owing to the toxicity of the benzene based structures [38], cholesterol and guanosine conjugated ligands of gatifloxacin and moxifloxacin are considered to be a lead structures for further testing in the wet laboratory.

## List of abbreviations

S: Sensitive; R (L): Low level resistant; R (M): Medium level resistant; R (H): High level resistant

## Authors' contributions

CNP, MD and SS designed the methods and experimental setup. SS and ARS carried out the implementation of the various methods. SS and ARS wrote the manuscript and MD & CNP revised it critically. All the authors have read and approved the final manuscript.

## Supplementary Material

Additional file 1 **Figure S1**. Structures derived from Gatifloxacin and Moxifloxacin by altering the functional groups of 7^th ^(A) and 3^rd ^(B) positions of quinolone ring. The MFX or GFX moieties in the 7^th ^position for 3^rd ^position modifications are indicated. M.W - Molecular weight. Click here for file
