# Supplementary material for: Docking studies on novel analogues of 8 methoxy fluoroquinolones against GyrA mutants of Mycobacterium tuberculosis
Source: BMC Struct Biol. 2011 Dec 12;11:47. doi: 10.1186/1472-6807-11-47 (PMC3298726; doi:10.1186/1472-6807-11-47)
Supplement: Additional file 1 — Figure S1. Structures derived from Gatifloxacin and Moxifloxacin by altering the functional groups of 7th (A) and 3rd (B) positions of quinolone ring. The MFX or GFX moieties in the 7th position for 3rd position modifications are indicated. M.W - Molecular weight. [file 1472-6807-11-47-S1.PDF]

‘Antibacterial spectrum’ determining region modifications – A

S. No	Moiety and chemical features	S. No	Moiety and chemical features
1	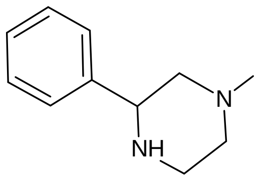 $C_{24}H_{24}FN_3O_4$ M.W: 437.463 LogP: 2.26±1.55	2	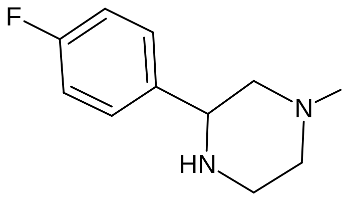 $C_{24}H_{23}F_2N_3O_4$ M.W: 455.453 LogP: 2.31±1.56
3	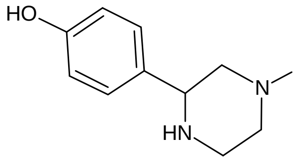 $C_{24}H_{24}FN_3O_5$ M.W: 453.462 LogP: 1.53±1.55	4	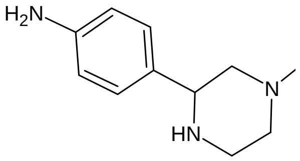 $C_{24}H_{25}FN_4O_4$ M.W: 452.478 LogP: 0.98±1.55
5	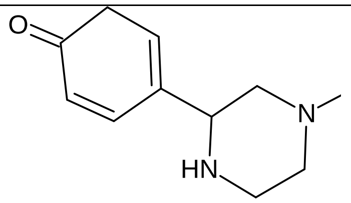 $C_{24}H_{24}FN_3O_5$ M.W: 453.462 LogP: 0.96±1.55	6	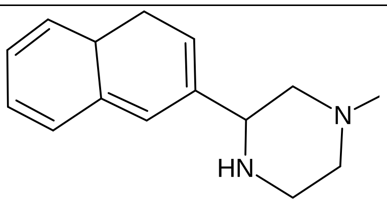 $C_{28}H_{28}FN_3O_4$ M.W: 489.538 LogP:3.76±1.55
7	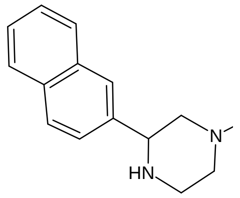 $C_{28}H_{26}FN_3O_4$ M.W: 487.522 LogP: 3.49±1.55	8	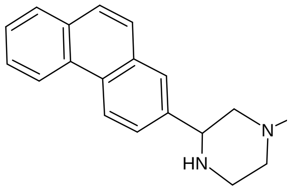 $C_{32}H_{28}FN_3O_4$ M.W: 537.580 LogP: 4.72±1.55

9	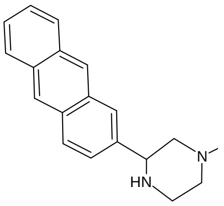 $C_{32}H_{28}FN_3O_4$ M.W: 537.580<br>LogP: 4.72±1.55	10	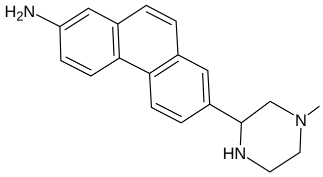 $C_{32}H_{29}FN_4O_4$ M.W: 552.595<br>LogP: 3.44±1.55
11	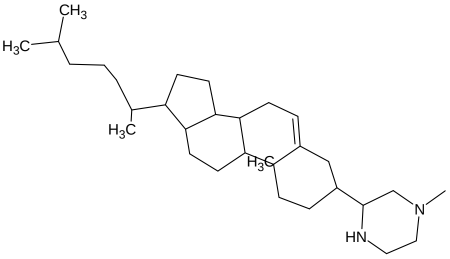 $C_{44}H_{62}FN_3O_4$ M.W: 715.979<br>LogP: 11.31±1.55	12	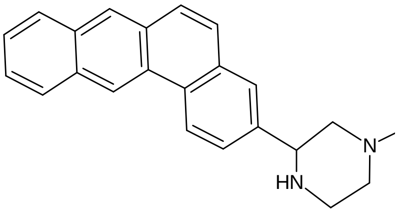 $C_{36}H_{30}FN_3O_4$ M.W: 587.639<br>LogP: 5.95±1.55
13	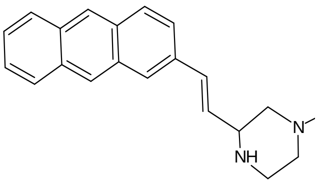 $C_{34}H_{30}FN_3O_4$ M.W: 563.618<br>LogP: 5.45±1.54	14	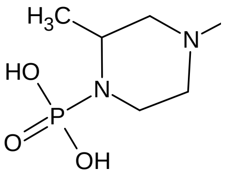 $C_{19}H_{23}FN_3O_7P$ M.W: 455.373<br>LogP: -1.17±1.55
15	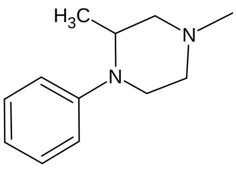 $C_{25}H_{26}FN_3O_4$ M.W: 451.490<br>LogP: 3.44±1.54	16	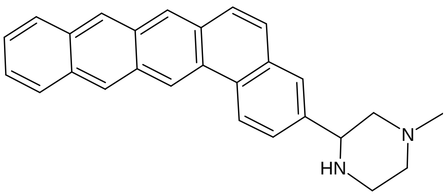 $C_{40}H_{32}FN_3O_4$ M.W: 637.698<br>LogP: 7.19±1.55

17	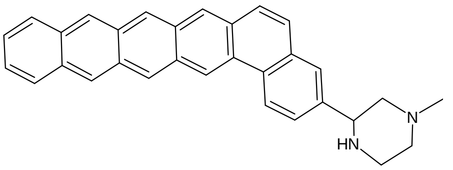<br>$C_{44}H_{34}FN_3O_4$ M.W: 687.756<br>LogP: 8.42±1.55	18	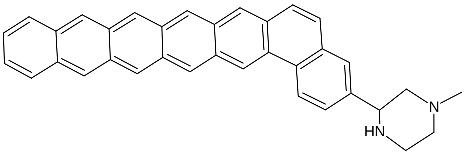<br>$C_{48}H_{36}FN_3O_4$ M.W: 737.815<br>LogP: 9.65±1.55
19	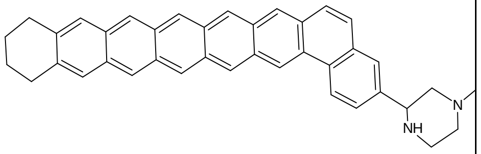<br>$C_{52}H_{42}FN_3O_4$ M.W: 791.905<br>LogP: 11.33±1.55	20	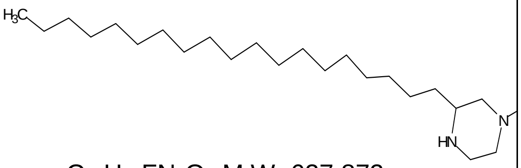<br>$C_{37}H_{58}FN_3O_4$ M.W: 627.872<br>LogP: 10.78±1.54
21	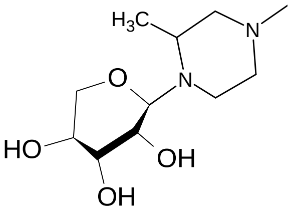<br>$C_{24}H_{30}FN_3O_8$ M.W: 507.508<br>LogP: 1.14±1.55	22	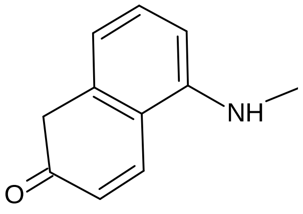<br>$C_{24}H_{19}FN_2O_5$ M.W. 434.415<br>LogP: 2.50+/- 1.54
23	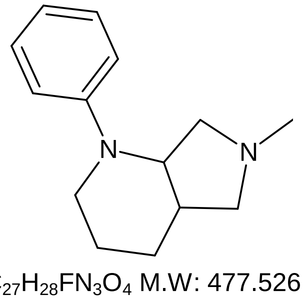<br>$C_{27}H_{28}FN_3O_4$ M.W: 477.526<br>Log P: 3.55+/- 1.54	24	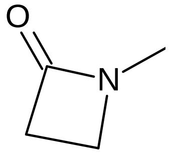<br>$C_{17}H_{15}FN_2O_5$ M.W: 346.309<br>LogP: 0.35±1.54

‘DNA gyrase binding’ region modifications – B

25	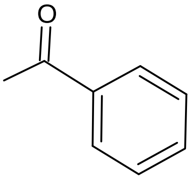<br>GFX<br>$C_{25}H_{26}FN_3O_3$ M.W: 435.490<br>LogP: 2.81±1.55	26	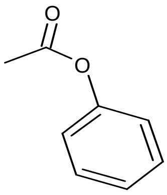<br>GFX<br>$C_{25}H_{26}FN_3O_4$ M.W: 451.490<br>LogP: 2.87±1.55
27	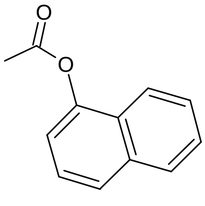<br>GFX<br>$C_{29}H_{28}FN_3O_4$ M.W: 501.548<br>LogP: 4.10±1.55	28	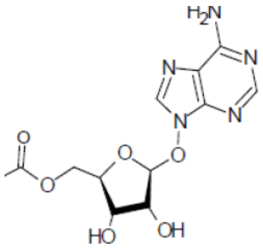<br>GFX<br>$C_{29}H_{33}FN_8O_7$ M.W: 624.620<br>LogP: 0.94+/- 1.57
29	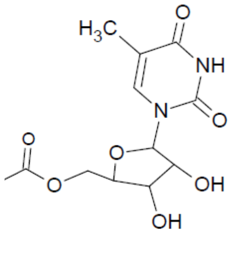<br>GFX<br>$C_{29}H_{34}FN_5O_9$ M.W.: 615.607<br>LogP: 0.16+/- 1.56	30	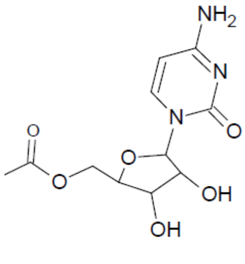<br>GFX<br>$C_{28}H_{33}FN_6O_8$ M.W: 600.594<br>LogP: -0.40+/- 1.56

31	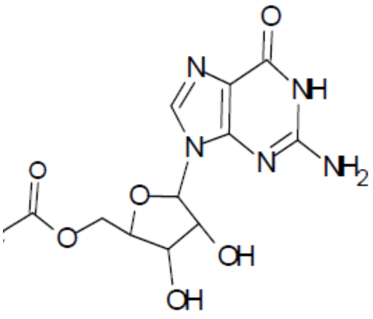<br>GFX<br>$C_{29}H_{33}FN_8O_8$ M.W: 640.620<br>LogP: 0.73+/- 1.57	32	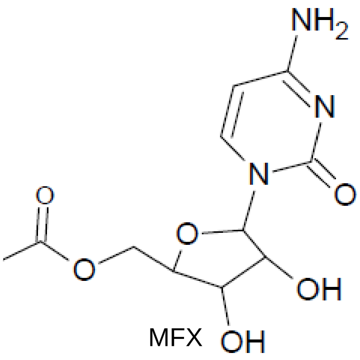<br>MFX<br>$C_{30}H_{35}FN_6O_8$ M.W: 626.633<br>LogP: -0.02+/- 1.56
33	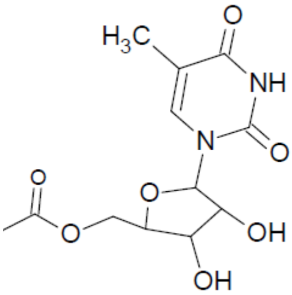<br>MFX<br>$C_{31}H_{36}FN_5O_9$ M.W: 641.643<br>LogP: 0.55+/- 1.56	34	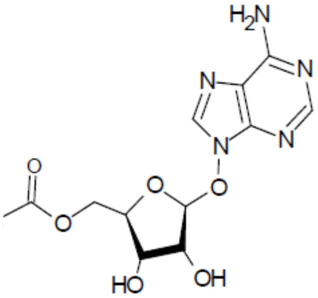<br>MFX<br>$C_{31}H_{35}FN_8O_8$ M.W. 666.657<br>LogP: 0.19+/- 1.62
35	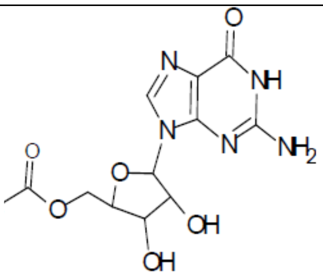<br>MFX<br>$C_{31}H_{35}FN_8O_8$ M.W: 666.657<br>LogP: 1.12+/- 1.57	36	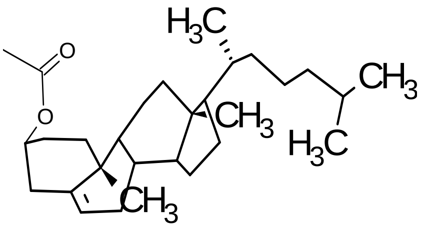<br>GFX<br>$C_{46}H_{66}FN_3O_4$ M.W: 744.032<br>LogP: 11.51±1.55

37	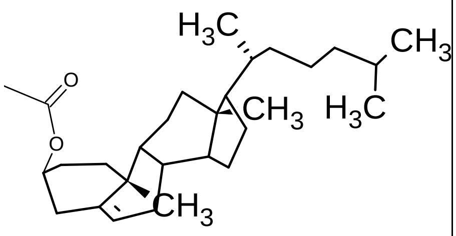<br>MFX<br>$C_{48}H_{70}FN_3O_4$ M.W: 772.085<br>LogP: 12.30+/- 1.54	38	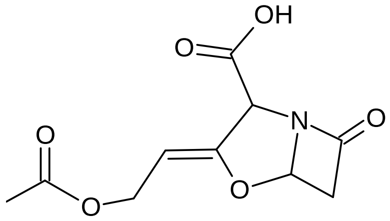<br>GFX<br>$C_{27}H_{29}FN_4O_8$ M.W: 556.539<br>LogP: -0.39±1.59
39	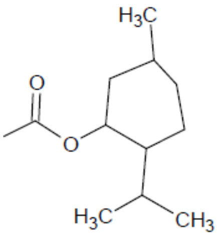<br>MFX<br>$C_{31}H_{42}FN_3O_4$ M.W: 539.681<br>LogP: 5.29±1.54		
